# Advanced nanomaterial for prostate cancer theranostics

**DOI:** 10.3389/fbioe.2022.1046234

**Published:** 2022-11-01

**Authors:** Bin Hao, Li Wei, Yusheng Cheng, Zhifang Ma, Jingyu Wang

**Affiliations:** ^1^ Department of Urology, Central Hospital, China Railway 17th Bureau Group Co., Ltd., Shanxi, China; ^2^ Internal Medicine, Rongjun Hospital of Shanxi Province, Shanxi, China; ^3^ Department of Urology, First Hospital of Shanxi Medical University, Shanxi, China; ^4^ College of Biomedical Engineering and Technology, Tianjin Medical University, Tianjin, China

**Keywords:** nanomaterial, prostate cancer, diagnosis, therapy, theranostics

## Abstract

Prostate cancer (PC) has the second highest incidence in men, according to global statistical data. The symptoms of PC in the early stage are not obvious, causing late diagnosis in most patients, which is the cause for missing the optimal treatment time. Thus, highly sensitive and precise early diagnosis methods are very important. Additionally, precise therapy regimens for good targeting and innocuous to the body are indispensable to treat cancer. This review first introduced two diagnosis methods, containing prostate-specific biomarkers detection and molecular imaging. Then, it recommended advanced therapy approaches, such as chemotherapy, gene therapy, and therapeutic nanomaterial. Afterward, we summarized the development of nanomaterial in PC, highlighting the importance of integration of diagnosis and therapy as the future direction against cancer.

## 1 Introduction

Prostate cancer (PC) has become the 4th most commonly diagnosed cancer (7.3% of all sites), according to GLOBOCAN 2020 estimates from the International Agency for Research on Cancer. For men, there is a higher incidence and mortality, which rank 2nd and 5th, respectively, among all cancers according to global statistics. In 2020, there were about 1.4 million new cases and estimated 375,000 deaths worldwide, which attracted increased attention to the theranostics of PC (Sung et al., 2021).

PC patients do not demonstrate evident symptoms in the early stage. Many of the symptoms are easily overlooked because of their similarity with the symptoms of prostatitis and benign prostatic hyperplasia. PC diagnosis, especially in the early phase, is indispensable for curing PC. Digital rectal examination and serum biomarker detection are the simplest non-invasive methods (Carter et al., 2013; Huang et al., 2022). Magnetic resonance imaging (MRI), computed tomography (CT), and ultrasound (US) are very common imaging methods (Carter et al., 2013; Huang et al., 2022). Aspiration biopsy is usually used as a gold standard to improve accuracy. The cure rate of PC is largely dependent on the PC stage at diagnosis. Treatment plans also rely on staging.

Surgery is a ubiquitous therapy that is almost always applied to malignant tumors. Androgen ablation is a unique method of PC treatment since this type of cancer is a hormone-dependent illness (Seidenfeld et al., 2000). In some PC stages, radiotherapy and chemotherapy are effectively employed to delay clinical metastasis and further progression. These diagnosis and treatment strategies induce a decrease in mortality rate, especially in high-income countries (Sung et al., 2021). Although these efforts are beginning to show results, there are still some issues, such as low diagnosis sensitivity, limited options of targeted drugs, and a high recurrence rate.

Nanomaterials, which have an enhanced permeability and retention (EPR) effect, are emerging as carriers or drug molecules to address the problems mentioned before( Li et al., 2020b; Li et al., 2022 ). This review focused on the frontiers of nanomaterials in PC theranostics. First, we discussed the diagnosis of PC, containing prostate-specific biomarkers detection and molecular imaging. Then, we provided some therapeutic approaches, of which chemotherapy is the most widespread one. Selecting appropriate targets and nanomaterials to reduce side effects and drug resistance is essential in cancer treatment. Gene therapy, containing polymers, membrane vesicles, etc., as the carriers, is used in certain cases. Therapeutic nanomaterials are also introduced, such as selenium nanoparticles, graphene, and targeting peptides. Lastly, we proposed that the integration of diagnosis and therapy should be a significant breakthrough in cancer management.

## 2 Diagnosis of PC

### 2.1 Prostate-specific biomarkers detection

Different biomarkers, such as serum, urine, and exosome biomarkers, are effectively used for cancer detection and screening (Carter et al., 2013; Salimi et al., 2013; Patra et al., 2015; Kowalczyk et al., 2022; Yan et al., 2022). Among these biomarkers, prostate-specific antigen (PSA) is the most common one used to diagnose PC. PSA is the biomarker for primary tumor diagnosis approved by the US Food and Drug Administration (FDA), and it has been generally employed to diagnose PC in the clinic (Sanders et al., 2014). Although efficient testing occurred due to the evidence of PC spike during the early 1990s, after its widespread uptake (Siegel et al., 2022), this method frequently provides false-positive and false-negative results and leads to overdiagnosis (Huang et al., 2022; Kshirsagar et al., 2022). More sensitive and accurate detection means are emerging to optimize PSA testing.

The most widely used method for detecting proteins is enzyme-linked immunosorbent assay (ELISA) (Patra et al., 2015; Sharafeldin et al., 2017). Besides, many other optimized methods can screen PSA, such as fluorescence resonance energy transfer (FRET) (Furukawa et al., 2016), electrochemical sensor (Salimi et al., 2013; Wei et al., 2018), electrochemiluminescence (ECL) (Khoshfetrat et al., 2019), localized surface plasmon resonance (LSPR) (Acimovic et al., 2014; Sanders et al., 2014), surface-enhanced Raman scattering (SERS) (Cheng et al., 2017), and some combined methods (Duan et al., 2018). Development of various approaches results in great improvement; for example, PSA can be evaluated not only in male but also in female serum, although the value of PSA in women is very limited, and PSA detection is difficult (Patra et al., 2015). In the review, we mainly concentrated on electrochemistry and SERS to diagnose PSA.

**FIGURE 1:**
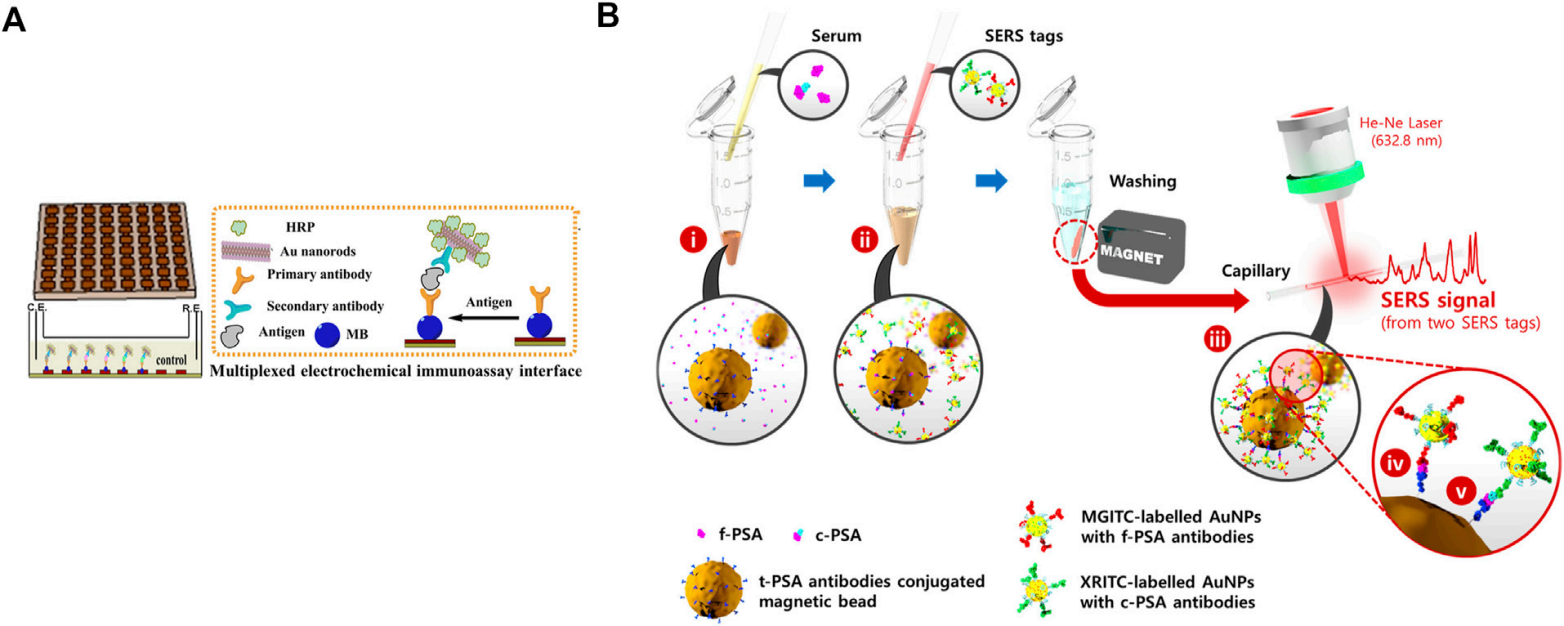
**(A)** Preparation of magnetic slice and multiplexed electrochemical immunoassay interface. Reprinted with permission from (Liu et al., 2014). Copyright 2014, American Chemical Society. **(B)** Preparation of t-PSA antibody-loaded beads, two characteristic SERS tags, and detection of two biomarkers. Reprinted with permission from (Cheng et al., 2017). Copyright 2017, American Chemical Society.

Timely medical interference and sensitive testing methods are necessary. As shown in Figure 1A, a polydimethylsiloxane (PDMS) slice is decorated with 8 × 8 nano-Au electrodes. The slice is then modified with the primary antibody by the magnetic force between the magnetic beads (MBs) and nano-Au electrodes. In the presence of PSA, which is to be detected, the three-in-one compounds, the second antibody-horse radish peroxidase (HRP)-Au nanorods, are added to bind with PSA. The reduction reaction of H_2_O_2_, the added substrate, triggers the generation of electrochemical signals, which can quantitatively evaluate PSA with good selectivity and appropriate detection limit. This assay provides a new direction to detect PSA more precisely and accurately (Liu et al., 2014).

Elevated serum PSA, specifically 4–10 ng ml^−1^, is not only associated with PC but also with benign prostate hyperplasia and prostatitis (Liu et al., 2014). Therefore, using only PSA to diagnose PC is insufficient. Prostate-specific membrane antigen (PSMA) is a protein of prostatic epithelial cell membrane with higher specificity (Liu et al., 2014; Sharafeldin et al., 2017). In a study, graphene oxide (GO) nanosheets were deposited with Fe_3_O_4_ nanoparticles (Fe_3_O_4_@GO). The first antibody was loaded onto the magnetic Fe_3_O_4_@GO composite by chemical crosslinking, which could specifically capture the biomarker. The biomarker-laden Fe_3_O_4_@GO was delivered into a detection chamber, which was coated with the second antibody. The interaction between the second antibody and the biomarker made an amperometric response after adding H_2_O_2,_ as catalyzed by Fe_3_O_4_@GO, which had peroxidase-like activity. This system could simultaneously detect PSA and PSMA, which might promote the sensitivity and preciseness of using biomarker-based diagnosis in the clinic (Sharafeldin et al., 2017).

Another biomarker to address the nonspecific expression of PSA in PC is free PSA (f-PSA). Most PSA, easily attaching to other proteins, exists in serum as a complex. For example, PSA and protease inhibitors create stable complexed PSA (c-PSA). In contrast, another PSA not binding to any proteins is known as f-PSA. According to a report, f-PSA decreases in men with PC compared with those with benign prostate hyperplasia or prostatitis (Cheng et al., 2017). Therefore, in clinical diagnosis, the decline in free to total PSA (t-PSA) ratio is employed to identify PC as an important index to distinguish PC and other benign diseases. As shown in Figure 1B, magnetic beads (MBs) are loaded with t-PSA antibodies, which can simultaneously bind to f-PSA and c-PSA. Two tags are prepared based on SERS, with conjugation of f-PSA and c-PSA antibodies, respectively. Adding SERS tags to the centrifuge tube forms sandwich complexes. The beads are separated by the magnetic bar, which produces SERS signals to quantify f-PSA and c-PSA according to characteristic Raman peaks of two different tags (Cheng et al., 2017). This easy-to-perform and accurate approach might help develop new methods for the primary diagnosis of PC.

### 2.2 Molecular imaging

Molecular imaging, such as MRI and CT (Hurley et al., 2016; Schilham et al., 2021), can also diagnose diseases. Additionally, the preparation of aspiration biopsy may depend on CT or transrectal ultrasound (TRUS) (Logan et al., 2014). MRI, which avoids ionizing radiation exposure, is more acceptable. This technology was developed several decades ago. MRI usually uses a contrast agent (CA) as the adjuvant to increase sensitivity and improve imaging quality. Basically, CA is divided into two categories: paramagnetic and superparamagnetic. Paramagnetic agent, such as gadolinium (Gd), which can increase signals, is also named positive CA. In contrast, the superparamagnetic one, commonly called negative CA, has the effect of decreasing signals to enhance contrast. Iron oxide is representative of this type (Liu et al., 2021b; Jiang et al., 2021). There are still some issues with MRI. For example, retention of CA is limited for quick body clearance, affecting imaging quality and accuracy. In recent years, many different categories of nanomaterials have been applied in MRI, qualitatively enhancing its specificity and sensitivity.

Yang et al. (2015) have reported a nanomaterial based on CA. Graphene oxide (GO) nanosheets were grafted with dendrimers (DEN), and then gadolinium diethylene triamine pentaacetate (Gd-DTPA) and prostate stem cell antigen (PSCA) monoclonal antibody (mAb) were sequentially added (GO-DEN [Gd-DTPA]-mAb). GO-DEN (Gd-DTPA)-mAb could target PSCA overexpressing cells (PC3), inducing enhancement of T1-weighted contrast. In *vivo* experiment, after 1 h or 4 h of intravenous injection, compared with GO-DEN (Gd-DTPA), GO-DEN (Gd-DTPA)-mAb triggered a more obvious increase in the image signal to PC3 tumor (Guo et al., 2016). This work was based on the interaction between antigen and antibody, implying the promise of targeting nanomaterials in MRI and other imaging modalities.

## 3 PC therapy

### 3.1 Chemotherapy

Chemotherapy originated during a war when scientists accidentally found that nitrogen mustard played a role in inhibiting lymphoma (Rahmani and Abdollahi, 2017). Now, it is frequently employed for advanced/metastatic cancer or after surgery to prevent relapse (Adahoun et al., 2017). According to the source and mechanism, chemotherapeutics categorize into different types, such as alkylating agents (Liang et al., 2018), antimetabolites (Ren et al., 2014), compounds extracted from plants (Zhang et al., 2018), metals (Cai et al., 2017). One group of these drugs, which is extracted from plants, comprises nature molecules and serves as a very promising and safe choice to treat cancers.

**FIGURE 2:**
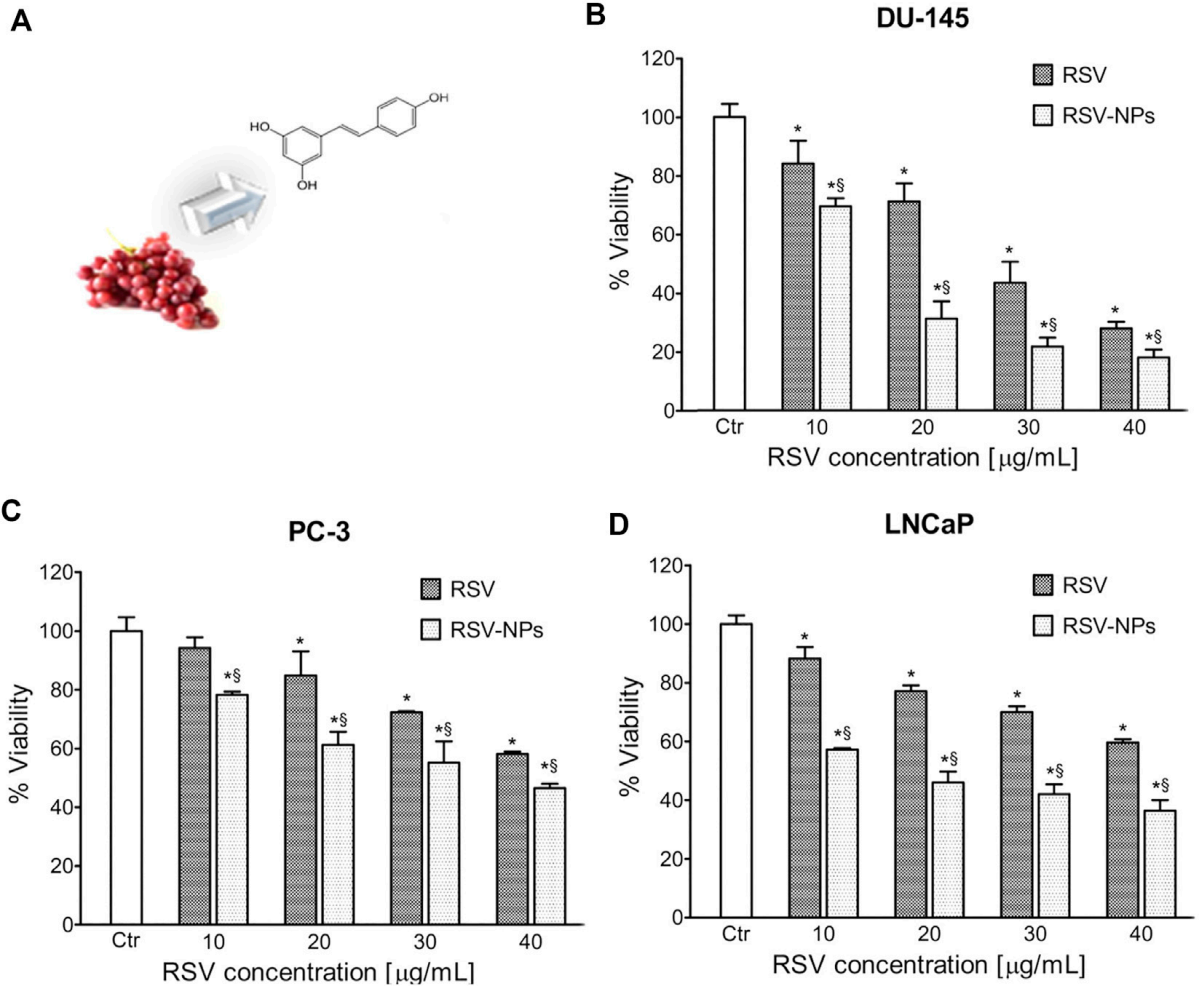
**(A)** Extraction of RSV from grapes and molecular structure of RSV. Toxicity of RSV/RSV-NPs to different cell lines: **(B)** DU-145, **(C)** PC-3, and **(D)** LNCaP. Reprinted with permission from (Sanna et al., 2013). Copyright 2013, American Chemical Society.

As shown in Figure 2A, trans-resveratrol (RSV), present in multiple plants, such as grapes and peanuts, has good antiaging and antitumor effects. Nevertheless, this molecule has some drawbacks also commonly found in other antitumoral drugs, such as poor solubility and bioavailability, instability, and low intracellular penetration. Polymeric nanoparticles (NPs), composed of a mix of poly (epsilon-caprolactone) (PCL) and poly (D,L-lactic-co-glycolic acid)-poly (ethylene glycol) conjugate (PLGA-PEG-COOH), can overcome this challenge. With NPs as the carrier, this system exhibits high RSV loading ability, controlled RSV release, and enhanced toxicity in all three PC cell lines compared to free RSV (Figures 2B,D) (Sanna et al., 2013). This system, addressing the issues that exist in RSV, might be further applied to other natural drugs.

Although these drugs are effective in cancer cells, other indexes, such as the biocompatibility to body index, also need evaluation. There is the nanomaterial based on genistein. Genistein (5, 7-dihydroxy-3-[-4-hydroxyphenyl]-4H-1-benzopyran-4-one) is also a natural molecule present in Leguminosae family, particularly in soy. Soy genistein (Gen) has proved to reduce the proliferation of PC cells (Vodnik et al., 2021). Due to the limitation of chemotherapy for its poor solubility and retention, which is similar to RSV, two variants of genistein-gold conjugates are provided: Gen@AuNPs1 and Gen@AuNPs2. The two nanoparticles are classified by different concentrations of Gen and Au^3+^. This system shows toxicity to all three PC cells (LNCaP, DU145, and PC3). More importantly, the nanoparticles maintain low toxicity to noncancerous cells (MRC-5, primary human cells) (Vodnik et al., 2021). This work highlights Au nanoparticles as the carrier to inhibit cancer cells and protect normal ones, broadening the applications of nanomaterial.

Nanomaterial is widely used for its excellent performance, but the addition of extra substances into natural drugs increases its unsafety. There is an intriguing approach to prepare a nanodrug (Bhawana et al., 2011). Curcumin ([E, E]-1,7-bis [4-hydroxy-3-methoxy-phenyl]-1,6-heptadiene-3,5-ione) (CUR) is another natural drug derived from rhizome of *Curcuma longa*. It is described as an antitumor, anti-inflammatory, and antimicrobial compound. In a study, the authors used a wet-milling technique to form CUR nanoparticles (nanocurcumin), which displayed good solubility and bioavailability. Nanocurcumin showed enhanced antitumor activity compared with parent CUR. Moreover, it had low toxicity to normal cells (HEK, human embryonic kidney cell line) (Adahoun et al., 2017). This work provided an innovative direction to improve the safety of nanomaterial delivery systems, motivating scientists to study other nanodrugs besides CUR.

Although an increasing number of natural drugs have been found, while some, for example, taxol, have been used as the first-line anticancer drugs, there still are some general problems. These include the disadvantage of all anticancer drugs used in the clinic. It is inevitable to induce side effects after chemotherapy; for example, patients suffer from vomiting, loss of appetite, and diarrhea (Zhang et al., 2022), making it a frightening therapy. Additionally, drug and even multidrug resistance (MDR) can be easily developed. Concentrating on these issues, the topical subjects are the selection of a target and using nanomaterial to reduce adverse effects and overcome drug resistance.

#### 3.1.1 Selection of a target to reduce side effects

**FIGURE 3:**
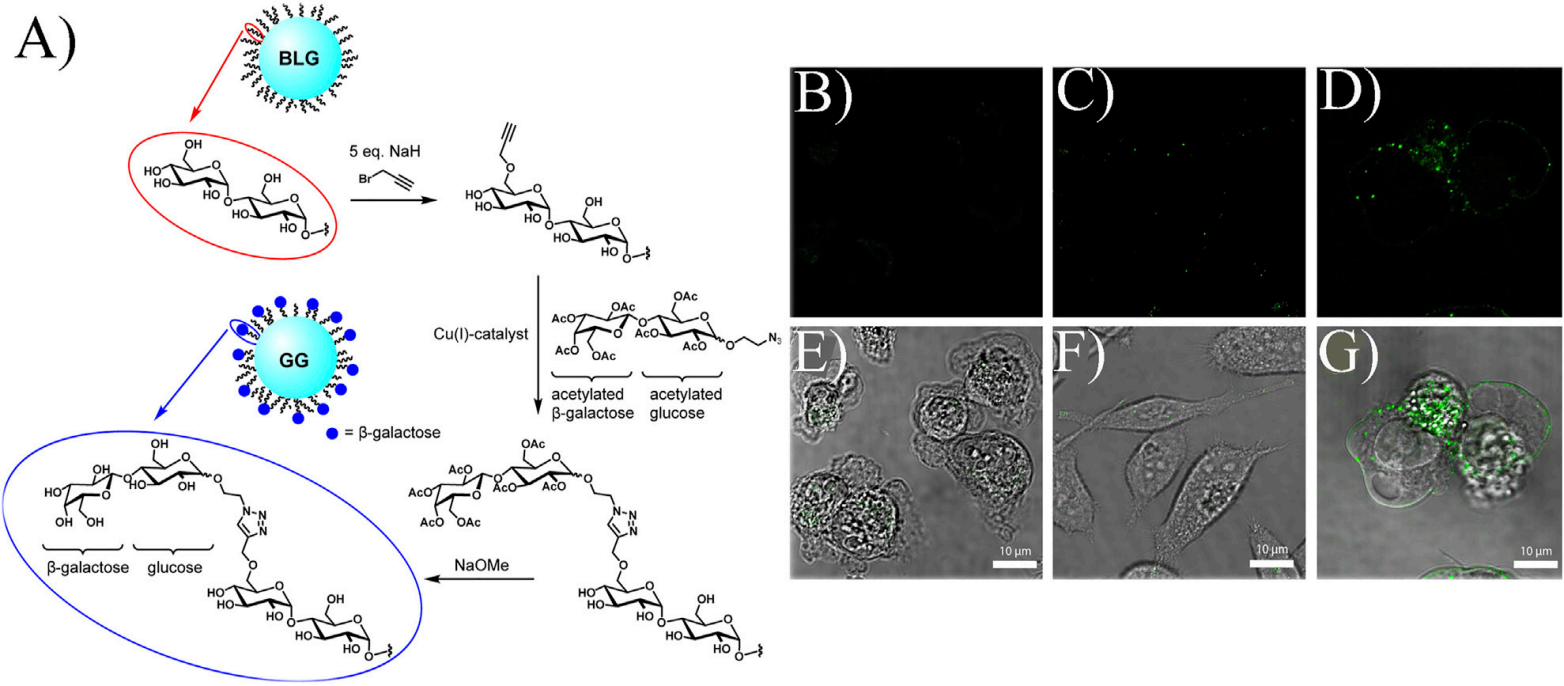
**(A)** Formation of GG. Confocal microscopy and bright field images of different compounds: **(B,E)** control, **(C,F)** FITC-labeled BLG, **(D,G)** FITC-labeled GG incubating PC cells. Reprinted with permission from (Besford et al., 2017). Copyright 2017, American Chemical Society.

The key issue in overcoming side effects is how to deliver drugs directly to tumor sites rather than to the whole body. The first question is how to select an effective target (Tong et al., 2010; Al-Mansoori et al., 2021). Galectin-1, a galactoside binding lectin, is upregulated in PC cells during disease development. Hence, galectin-1 is usually employed as the target to specifically inhibit PC cell survival. In one study, glyconanoparticles were used as they are more likely to bind lectin (Besford et al., 2017). As shown in Figure 3A, the bovine liver glycogen (BLG) nanoparticle is a branched polymer of glucose. After a series of chemical reactions, terminal galactoside is linked to BLG, named galactoside-glycogen (GG). This is a kind of nanoparticle that can specifically interact with galectin-1. To prove this point, fluorescein isothiocyanate (FITC, a fluorescent dye)-labeled BLG and GG were used. As shown in Figures 3B,G, compared with BLG, the fluorescence of GG was stronger, indicating that the improved nanoparticles had better performance in specifically binding to the PC cell membrane (Besford et al., 2017). This work selected galectin-1 as the target, proving that the biomarkers are indispensable in tumor management.

Although efficient, targeting agents have some problems. For instance, since the transferrin receptor (TfR) is overexpressed in numerous types of cancer cells, transferrin (Tf), which can specifically target TfR, is usually used as a targeting agent. However, it easily dissociates from TfR after losing an iron molecule in cells. In a study, a Tf variant (oxalate Tf) was adopted. Doxorubicin (DOX)-loaded poly (lactide-co-glycolide) (PLGA) nanoparticles (DPs), encapsulating Tf/oxalate Tf and polyethylene glycol (PEG), were the targeting system named Tf-PEG-DPs (TPDPs). It was then incorporated into a three-dimensional PLGA network, forming the system to release drugs controllably. Compared with the native TPDPs, the one containing oxalate was more effective in reducing the survival of PC cells (Lopes et al., 2017). This was the first example of using oxalate Tf in the PLGA network to enhance the inhibition effect against PC, which might promote the development of other targeting systems.

#### 3.1.2 Selection of nanomaterial to reduce adverse effects

**FIGURE 4:**
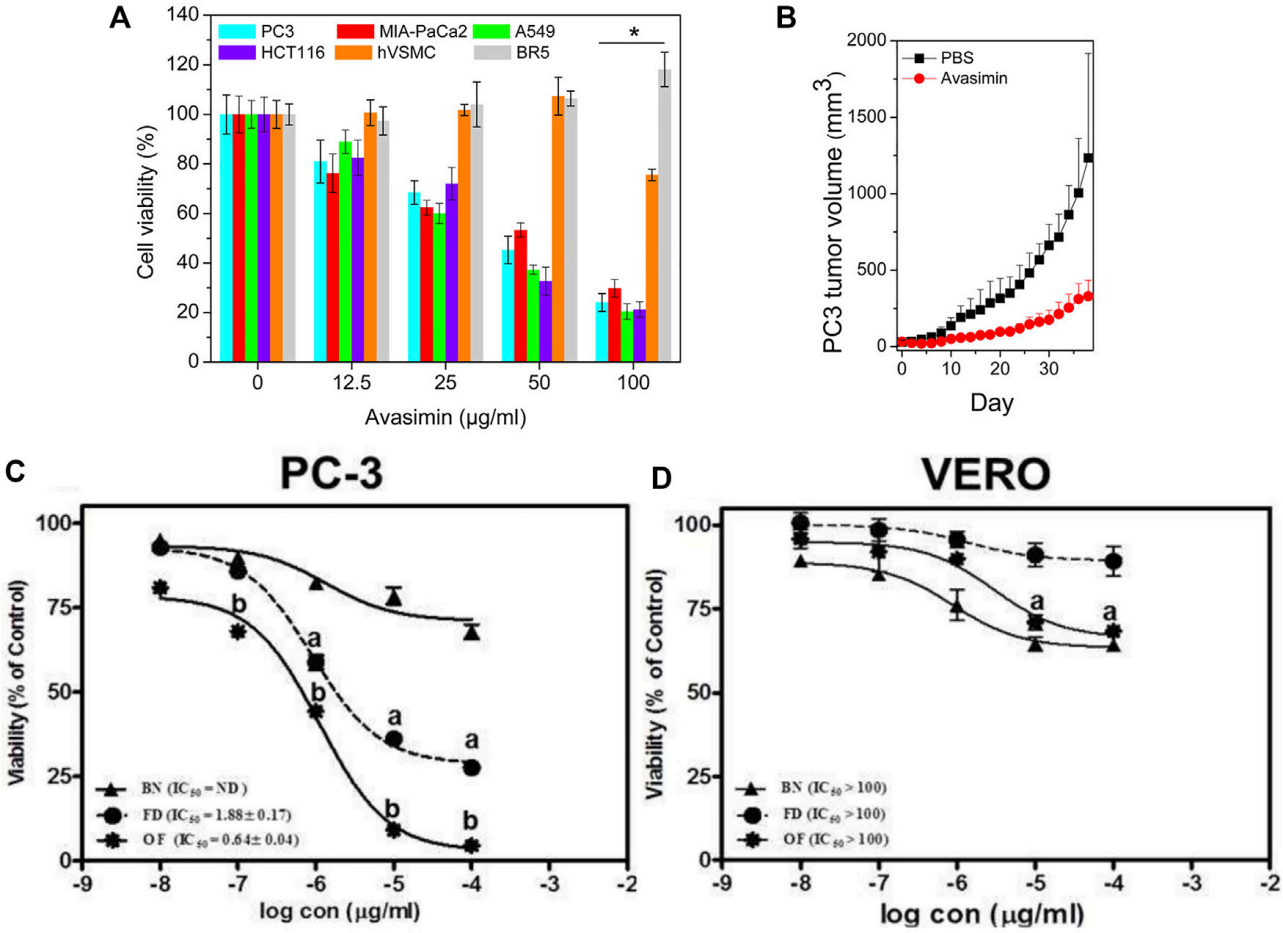
**(A)** Cell viability after treatment of avasimin in various cells. **(B)** PC3 tumor volume after the treatment with avasimin. Reprinted with permission from (Lee et al., 2015). Copyright 2015, American Chemical Society. Viability after the treatment with three substances in different cell lines: **(C)** PC3; **(D)** VERO. BN: blank niosomes, FD: Free drug. Reproduced with permission from (Ali et al., 2021).

The selection of a target is indispensable to a specific controlled drug release system. However, the choice of nanomaterial is more important (Shin et al., 2014). The nanomaterial requires good biocompatibility, appropriate rate of release and degradation, easy synthesis, etc. Avasimibe is an antitumor agent that works in many kinds of cancers. However, it has low solubility in water, inducing poor bioavailability in blood circulation. In a study, human serum albumin (HSA) was used to conjugate avasimibe, forming water-soluble nanoparticles named avasimin (Lee et al., 2015). As shown in Figure 4A, avasimin played a role in high PC3 inhibition and non-toxicity to normal cells (BR5: dermal fibroblast), showing excellent biocompatibility. The same results happened in *vivo* experiment, as shown in Figure 4B. H&E staining showed that there was no harm to the vital organs (Lee et al., 2015). These results verified the importance of nanomaterials regarding good performance.

Some drugs are very promising and even going into clinical trials. We should focus not only on these drugs but also drugs in suspension. For example, ZSTK474 (ZSTK) is an anticancer drug, especially against advanced cancer. Although it can inhibit phosphatidylinositol 3-kinase (PI3K), which is over-activated in PC, it has been withdrawn in the early clinical investigation because it activates macrophage polarization. Macrophages are very important in the tumor microenvironment. They can be polarized into two types: classical macrophage (M1) and alternative macrophage (M2). M1 inhibits cancers by activating the immune system. However, M2 plays the role in surviving tumor cells. Indomethacin (IND), an anti-inflammatory drug, can combine with ZSTK due to its reduction effect on M1 to M2 polarization, which enhances the immune response. Nevertheless, the issue of toxic ZSTK affecting normal tissues has not been solved. Hence, adding nanoparticles to this drug is necessary. A cancer cell membrane can be considered as a natural nanoparticle, and it can specifically target cancer cells and escape body clearance, thus, stabilizing the particle in long blood circulation. However, the source of the cell membrane is limited; therefore, it is necessary to search for an alternative. In a study, ZSTK was initially synthesized into nanoparticles named ZNPs. Then, IND was encapsulated in a liposome, which is a commercial agent similar to the cellular membrane but more accessible. Hybridizing the PC3 cracked cell membrane in IND/liposome generated I@CML. Afterward, ZNPs were decorated with I@CML, termed ZNPs/I@CML, to form the drug release system. Concrete experimental data *in vivo* and *in vitro* were shown, confirming the speculation of M1 creation and the higher inhibitory activity of ZNPs/I@CML in PC3 tumor mice compared with free Z + I and ZNPs/I@Lip. Additionally, H&E staining showed no obvious tissue damage and proved the biosafety of ZNPs/I@CML (Zhang et al., 2022). This system utilized the advantage of ZSTK, IND, and cellular membrane to inhibit PC cells, highlighting the importance of appropriate nanomaterial in optimizing therapeutic drugs.

Some cancers, such as PC and breast cancer, are different in their hormone dependence. Hence, antihormone drugs attract more attention. Flutamide (FLT) is currently a clinical drug for PC therapy. However, its poor solubility, low bioavailability, and short half-life (5–6 h) affect the therapy. Increasing the dose from 250 mg to 750 mg per day worsens FLT side effects, such as hepatotoxicity and a decrease in sexual desire. Optimizing nanomaterial containing particle size and surface charge thus is indispensable. In a study, FLT-loaded optimized niosomes (optimized formula, OF) were prepared. This system had reasonable drug release and a good inhibitory effect on PC. As shown in Figures 4C,D, compared to PC3, green monkey epithelial kidney cells (VERO) were almost not sensitive to OF (Ali et al., 2021). This was an example of nanovesicle optimization as the carrier of antiandrogen drugs, and it might be a promising method to optimize other antihormone drugs, such as estrogen.

#### 3.1.3 Overcoming drug resistance

Drug resistance is common in patients receiving chemotherapy for a long time. Glycolysisgenerally occurs in the absence of oxygen. Tumor cells have a rapid metabolic rate and consume large amounts of glucose. Hence, they generate different metabolic characteristics, such as aerobic glycolysis, which was first observed by Otto Warburg and was named the Warburg effect. Therefore, aerobic glycolysis can be employed in cancer therapy. Pyruvate kinase muscle isoform 2 (PKM2) is a very important enzyme converting phosphoenolpyruvate to pyruvate in aerobic glycolysis. Additionally, the dimers type, not the tetramers type, of PKM2 can function in this kind of glucose metabolism. Therefore, PKM2 activators, which transfer PKM2 dimers to its tetramers, have the potency to prevent the Warburg effect and promote tumor inhibition. Serine (Ser) is this kind of activators, but the effect is not sufficient. O-GlcNAcase (OGA), which is overexpressed in many malignant tumors, can improve the proliferation of tumor cells by inhibiting PKM2. In a study, nanomaterial based on OGA was designed: S (GlcNAc)-K (TPA-1) LVFF termed GPNA1. It contained four motifs: 1) ser (β-N-acetylglucosamine) (S [GlcNAc]) motif containing PKM2 activator and OGA targeting agent, 2) Lys (K) motif as the linker, 3) TPA-1 as the fluorescent molecule 4) Leu-Val-Phe-Phe (LVFF) motif for self-assembly. GPNA1 first formed nanoparticles in the water. After it specifically targeted cancer cells, upregulated OGA catalyzed GlcNAc removal, exposing Ser. The nano-activator was transformed from nanoparticles to nanofibers, promoting the formation of PKM2 tetramers and inhibiting tumor metabolism. Compared with GPNA2, GPNA1 showed higher toxicity to highly metastatic PC cell lines (PC-3M IE8 cells). GPNA2 was similar to GPNA1 but had Ala-Ala-Gly-Gly (AAGG) peptide to substitute the self-assembly motif, LVFF. Additionally, compared with GPNA2, GPNA1 could sensitize cells to docetaxel (DTX), overcoming chemotherapy resistance, in *vitro* and *in vivo* experiments (Hou et al., 2022). This project used enzymes as the target to prevent tumor metabolism, highlighting the function of self-assembly peptides against chemotherapy resistance.

Docetaxel (DTX), which can disturb mitosis in the cell cycle to induce cell death, is the standard chemotherapy in advanced PC (Bharali et al., 2017). However, the side effect and drug resistance are inevitable, as shown in clinical trials. Curcumin (CUR), a natural drug, has been shown to affect PC. Furthermore, many molecules that explain the inhibitory effect, such as nuclear factor (NF)-κB and p53, are relevant to DTX resistance. Hence, CUR can prevent DTX resistance and reduce PC cell survival. In a study, the lipid-polymer nanoparticles were synthesized, containing PLGA, lecithin, and PEG-DSPE (LPNs). LPNs were conjugated with DTX and CUR, termed DTX-CUR-LPNs. PLGA nanoparticles without lecithin and PEG-DSPE were used as a control to encapsulate DTX and CUR, named DTX-CUR-NPs. The *in vivo* antitumor efficacy was evaluated. Compared with LPNs, free CUR, free DTX, CUR-LPNs, DTX-LPNs, DTX-CUR-NPs, and DTX-CUR-LPNs exhibited the highest activity against PC, proving the synergistic effect of DTX and CUR (Yan et al., 2016). This was an example of effective combined drugs, paving the way for a double drug release system to prevent tumor progression.

### 3.2 Gene therapy

Gene therapy is usually used in tumors and monogenic diseases. It has achieved significant progress, such as in the treatment of hemophilia and leukemia (Nathwani et al., 2014; Kuehn, 2017). Many therapeutic regimens have been approved, such as Zynteglo, authorized by European Union for treating β-thalassemia (Harrison, 2019) and Luxturna (Darrow, 2019) and Kymriah (The, 2017), permitted by the FDA for the cure of genetic eye disease and acute lymphocytic leukemia, respectively. Although it has been developing for many years and many patients have been cured, there are still some failures, such as the death of an American boy, Jesse Gelsinger (Lehrman, 1999), and phase II clinical failure of a drug for heart failure, CUPID2 (Ratner, 2015).

There are some reasons for the mentioned failures, such as inappropriate vectors and limited target-gene expression. As the vector of a target gene, viruses are the most widely used for their high transfection efficacy. Nonetheless, a virus has non-negligible safety problems. For example, in 2000, after the treatment of severe combined immunodeficiency disease (SCID) by a retrovirus, some patients had genetic mutations and symptoms similar to those of leukemia (Check, 2003). Adenovirus can lower this risk because it functions not necessarily to insert its genome into the host genome. However, the immunogenicity of adenovirus is too high (Lehrman, 1999). According to the research, adeno-associated virus (AAV) has become a very promising vector for its low immunogenicity, but its capacity is poor, usually less than 5 kb. Meanwhile, although the virus vector has been remodified to delete a pathogenic gene, uncertain genetic recombination still exists in virus packing, increasing the risk of causing an illness. Some physical methods, which are not dependent on the carrier, usually need very expensive equipment, and they are generally harmful to cells, causing the limitation of wide employment. Therefore, a non-viral vector is emerging to overcome these drawbacks.

Non-viral vector is currently in the basic research phase. This kind of vector does not necessarily insert its genetic material into the host genome, which is relatively safe. Additionally, the length of the loaded gene is not restricted. There are many non-viral vector types, such as mesoporous silicon (Wang et al., 2020), metal nanoparticles (Peng et al., 2018), linear and dendritic polymers (Han et al., 2018), liposome and its derivatives (Fenton et al., 2017), proteins (Ping et al., 2017), and peptides and their derivatives (Guan et al., 2019). The research on non-viral vectors is not limited to *in vitro* experiments, and some *in vivo* experiments have had a very good effect. Therefore, the non-viral vector is developing for practical applications and has a remarkable efficiency in some diseases, such as eye disease (Jiang et al., 2019), degenerative disease, and cancer (Guo et al., 2012; Liu et al., 2019).

The most widely used commercial transfection agent is polyethylenimine (PEI). However, PEI has no targeting motif, limiting its applications. Therefore, there are large amounts of PEI derivatives to enhance transfection efficacy. There are some reports about the usage of two receptors of some viruses for uptake into host cells. Hence, simultaneously targeting two receptors to mimic a virus is a clever design. In a study, B6 peptide (Ac-CGHKAKGPRKNH2) was employed to substitute transferrin (Tf), a protein capable of increasing the interaction with tumor cells. Another peptide motif was RGD, well known for its facilitation of attaching to cells. In this work, PEI derivatives were designed, containing B6/RGD dual-targeted system, B6, or RGD single targeted system. Luciferase expression was used to evaluate the transfection efficiency. The dual-targeted polyplex showed almost equal efficacy compared to PEI in two PC cells and higher efficacy than any of the single systems (Nie et al., 2011). This work provided a dual-targeted system of gene therapy, paving the way to find more effective transfection agents.

Small interfering RNA (siRNA), which knockdowns the complementary mRNA to regulate gene expression, has been developed in the cancer therapy field ( Li et al., 2020a ). O’Driscoll et al. have reported a polymer nanocarrier to deliver siRNA. The compound of PEGylation of poly-L-lysine-cholic acid (PEG-PLL-CA) was designed. The linker between PEG and PLL was acid-sensitive, which made it cleavable in a tumor site. The release of PEG made it a more appropriate system to load drugs into cells. In this work, the vascular endothelial growth factor (VEGF) siRNA was used to inhibit tumor proliferation in transgenic adenocarcinoma of the mouse prostate cell (TRAMP C1) tumor mice. The efficacy of PEG-PLL-CA was comparable with JetPEI, a commercial *in vivo* PEI (Guo et al., 2012). This work showed siRNA encapsulated in polymer to inhibit tumors, which might stimulate the development of more efficient polymers, even surpassing the commercial transfection agents.

**FIGURE 5:**
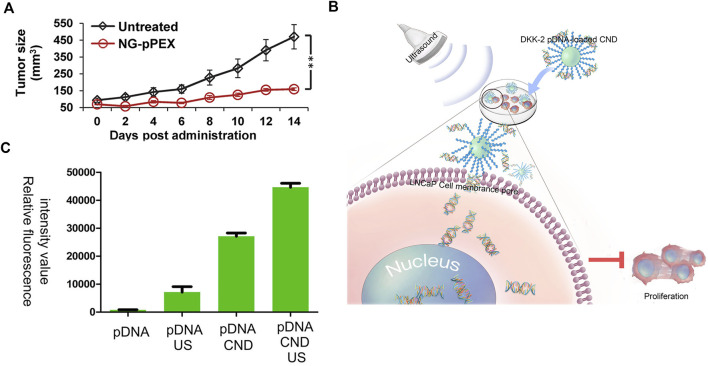
**(A)** Prostate tumor volume after the treatment with NG-pPEX using the untreated group as the control. Reprinted with permission from (Kaneti et al., 2016). Copyright 2016, American Chemical Society. **(B)** Schematic illustration of DKK-2 pDNA delivery into LNCaP cells. **(C)** Relative fluorescence intensity of pDNA/pDNA + US/pDNA + CND/pDNA + CND + US in LNCaP cells. Reproduced with permission from (Liu et al., 2021a).

Polymers, such as PEI, have the drawbacks of low transfection efficacy and high toxicity to cells. There are commercial substitutes, such as lipo2000 and lipo3000. In a study, membrane vesicles, which have the targeting capability, were used to deliver drugs for many diseases, such as cancer and HIV/AIDS. This system was termed nanoghosts (NGs). In this work, plasmid cDNA was used to encode the C-terminal motif of matrix metalloprotease-2, named the hemopexin-like domain (PEX). This kind of protein is toxic to cancer cells. As shown in Figure 5A, the tumor size after the treatment with NG-pPEX showed obvious shrink compared with control, proving its effective transfection (Kaneti et al., 2016). This work used innovative NGs as the vector, highlighting the development of membrane systems in gene therapy.

The above-discussed projects showed different carriers. Sometimes, there are other technologies added to enhance the transfection. A schematic illustration is shown in Figure 5B. The dickkopfs (DKKs) protein family is related to PC development. DKK-2 pDNA-loaded chitosan/perfluorohexane nanodroplets (CNDs) are added to the LNCaP cells, and then ultrasound is applied. The expression of DKKs protein prevents the proliferation of PC cells. Figure 5C exhibits the transfection effect of different groups, proving the importance of ultrasound (pDNA also encodes enhanced green fluorescent protein [EGFP] to evaluate transfection efficacy) (Liu et al., 2021a). This approach, dependent on ultrasound, is innovative and might be used in PC therapy in further clinical research.

### 3.3 Therapeutic nanomaterial

Nanomaterial functions not only as a carrier but also as a drug. Some nanomaterials are toxic to cancer cells, specifically inhibiting tumor proliferation. The trace element selenium can prevent cancer, and selenium nanoparticles (Nano-Se) are safer than other selenium compounds, such as sodium selenite and selenomethionine. Gao et al. have reported the function of Nano-Se. It showed the inhibitory activity of LNCaP cells, proving the potential of selenium nanomaterial in cancer therapy (Kong et al., 2011).

It is well known that metastasis of tumors is an issue in cancer therapy, dramatically shortening patients’ lives. Wei et al. have reported the function of graphene in tumor migration and invasion. In their work, graphene (Gra) and graphene oxide (GO) were used to suppress three kinds of cancer cells (MDA-MB-231: human breast cancer cell line, PC3, and B16F10: mouse melanoma cell line). The inhibitory activity was dose-dependent, and 90% of cells were viable after the treatment with Gra or GO at 20 μg/ml for 24 h. Nonetheless, under this condition, migration and invasion were prevented compared with control (Zhou et al., 2014). This work proved the function of Gra and GO in tumor migration and invasion, providing innovative insight into future cancer therapy.

**FIGURE 6:**
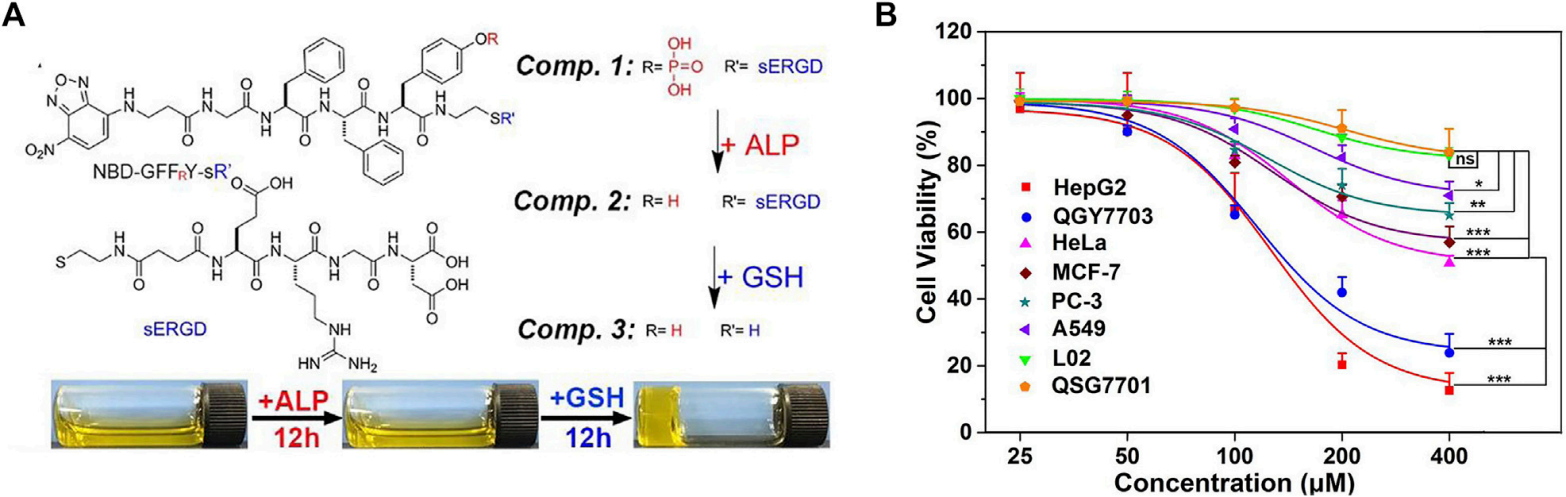
**(A)** Molecular structures of compounds 1, 2, and 3, tandem self-assembly formation, and a photograph after adding ALP and GSH to compound 1. **(B)** Cell viability after the treatment with different concentrations of compound 1 for 48 h of six cancer cells and two normal cells. Reproduced with permission from (Zhan et al., 2018)

Targeting agents are very indispensable in designing functional nanomaterial. Self-assembled peptides are very useful for inhibiting pathogenic cells, such as tumor cells and bacteria, and maintaining the physiological functions of normal tissues (Wang et al., 2021). In a study, tandem self-assembled peptide systems based on the targets were designed. As shown in Figure 6A, compound 1 contained four motifs: 1) NBD as the capped fluorescent motif, 2) GFF_R_Y as the self-assembly and alkaline phosphatase (ALP)-targeting motif, 3) ss as the glutathione (GSH) targeting motif, 4) RGD as the targeting motif to cancer cells. Adding ALP to compound 1 formed compound 2, which was a nanoparticle imaged by a transmission electron microscope (TEM) scan. By further using GSH, compound 3 was formed, generating the hydrogel. TEM image showed the nanofibers in the yellow hydrogel. Some cell experiments were conducted to further prove the tandem self-assembly. In many cancer cells, ALP and GSH are overexpressed. Nanoparticle formation by ALP catalysis and further formation of nanofibers by GSH cleavage in liver cancer cells had been evidenced by confocal laser scanning microscopy. This tandem self-assembly system was toxic to various cancer cells containing PC3 but was almost innocuous to normal liver cells, LO2 and QSG7701, as shown in Figure 6B (Zhan et al., 2018). Although this system had a less inhibitory effect on PC cells than liver cancer ones, it provided the theoretical and experimental basis for tandem self-assembly, which might benefit the development of more efficient nanomaterials in future research on PC.

This review mainly introduced chemotherapy, gene therapy, and therapeutic nanomaterial. Besides these methods, there are some other PC therapies, such as photodynamic therapy (PDT) (Lin et al., 2016), sonodynamic therapy (SDT) (Hadi et al., 2021), and combined therapy (Lee et al., 2011; Min et al., 2017; Mirjolet et al., 2017). Immunotherapy is effective in many diseases, such as allergic rhinitis (Liu et al., 2015), diabetes (Bluestone et al., 2015), HIV infection (Caskey et al., 2015), and especially cancer (Kanapathipillai et al., 2012; Hu et al., 2015; Stephan et al., 2015). Immunotherapy combination with other therapies, such as chemotherapy, is very popular and efficient. Jon et al. have reported a delivery system based on chemoimmunotherapy. CpG is an oligonucleotide that can stimulate the immune system. CpG-dendrimer conjugate was used as the carrier to load doxorubicin (Dox), an antitumor drug in the clinic. This system showed more effective antitumor activity than free Dox with the same dose in the 22RV1 (a kind of PC cell line) tumor model (Lee et al., 2011). Chemotherapy can combine not only with immune treatment but also with radiotherapy. Docetaxel (DTX) can sensitize cells to radiotherapy, but side effects and drug resistance limit its applications. There was a report based on titanate nanotubes (TiONts) to solve this challenge. This nano-carrier-loaded docetaxel (TiONts-DTX) enhanced the radiosensitivity. The effect of radiotherapy correlated with TiONts-DTX was assessed in PC3 tumor mice. The dual therapy system had stronger inhibitory efficacy compared to single therapy (Mirjolet et al., 2017). These data confirmed the effect of chemotherapy with other treatments. Additionally, combined gene therapy has been reported. Gold nanorods (GNR) were decorated with dipicolyl amine (DPA), which could bind Zn^2+^ cations, forming Zn (II)/DPA-GNR nanoparticles. They could deliver siRNA because of the interaction between Zn (II)/DPA and siRNA. More importantly, this system also enabled photothermal therapy, benefiting from GNR, exhibiting significant inhibitory activity in PC3 tumor mice (Min et al., 2017). In summary, a combined therapy system, which is superior to a single therapy, is a direction in future research and more applicable to individualized treatment.

## 4 Outlook

Most cases of PC are diagnosed at an advanced stage, III or IV, missing the best time to treat cancer. Aspiration biopsy is invasive to patients. Therefore, less invasive diagnosing methods in the early stage are requisite. Nanomaterial provides a strategy to enhance the sensitivity and accuracy of these methods. Traditional therapies for cancer include surgery, chemotherapy, and radiotherapy. However, some issues, such as side effects, MDR, metastasis, and relapse, limit the development of cancer therapy. Some innovative approaches based on nanotechnology are emerging, such as PDT and SDT. However, sometimes, the bioavailability of these drugs is still very low. Real-time detection and treatment become urgent needs. The word “theranostics” has been first introduced by John Funkhouser (Choudhury and Gupta, 2018). It means using one nanomaterial system to simultaneously diagnose and treat disease. For example, Ray et al. have reported a multifunctional gold nanoparticle system based on surface-enhanced Raman scattering (SERS). It had a triple function: diagnosis and treatment of PC cells and monitoring the photothermal regimen response (Lu et al., 2010). Therefore, integration of diagnosis and treatment may be another direction to overcome the barriers in PC therapy. Although most of these methods are in the basic research stage, they lay the foundation should the nanomaterial apply in the clinic in the future.

Although some anticancer nanomedicines have been applied in the clinic, such as Myocet, approved by European Medicines Agency (EMA) in 2000 to fight against breast cancer and DHP 107, authorized by South Korea in 2016 for gastric cancer therapy (He et al., 2019), there are still some disadvantages of nanomaterials, limiting their development. For example, the degradation and biodistribution of nanomaterial in*in vivo* are uncontrollable. Appropriate degradation rate and biodistribution of nanomaterial in *vivo* are necessary in the PC diagnosis and therapy. Therefore, precise design of nanomaterial is indispensable, containing charge, hydrophilia, size, configuration, and so on (Yang et al., 2015). To simplify the procedure, computer aided design is popularly used (Lai et al., 2017; Shao et al., 2020). Another common uncertain factor of nanomaterial used in *vivo* is the biocompatibility. Many of the nanomaterials are toxic to the vital organs or trigger the inflammatory response of the body, which makes us urgently establish an integrated evaluation system to screen nanomedicines (Heller et al., 2020). Although there are some difficulties in screening cancer nanomedicines, it is more attractive to scientists to study the characteristics of nanomaterial and further overcome the challenges (Sun et al., 2020).

Since the first nano-based cancer drug (Doxil) was approved by FDA in 1995, nanomedicines have been authorized in a few kinds of cancers therapies, such as ovarian and non-small-cell lung cancer, myeloma, osteogenic sarcoma (He et al., 2019; Zhao et al., 2022). There are a few PC nanomedicines employed in clinical phase Ⅱ, such as BIND-014, Tecemotide, and CT-2103 and clinical data shows the positive efficiency of BIND-014 (Autio et al., 2018; He et al., 2019). BIND-014 is a docetaxel nanoparticle which targets the prostate-specific membrane antigen (PSMA). Although PC, especially in the advanced period, can shorten the lives and cause the pain of patients, it is still hopeful by the effective clinical data. More importantly, the basic researches have been developing fast, and they might provide theoretical and experimental foundation for the clinical progress in the future.
